# Hyoid kinematic features for poor swallowing prognosis in patients with post-stroke dysphagia

**DOI:** 10.1038/s41598-020-80871-4

**Published:** 2021-01-14

**Authors:** Woo Hyung Lee, Min Hyuk Lim, Han Gil Seo, Byung-Mo Oh, Sungwan Kim

**Affiliations:** 1 Department of Rehabilitation Medicine, Seoul National University Hospital, Seoul National University College of Medicine, 101 Daehak-ro, Jongno-gu, Seoul, 03080 Republic of Korea; 2 Department of Biomedical Engineering, Seoul National University College of Medicine, 101 Daehak-ro, Jongno-gu, Seoul, 03080 Republic of Korea; 3 National Traffic Injury Rehabilitation Hospital, Yangpyeong, Gyeonggi-do 12564 Republic of Korea; 4 Institute of Aging, Seoul National University, 1 Gwanak-ro, Gwanak-gu, Seoul, 08826 Republic of Korea; 5 Neuroscience Research Institute, Seoul National University College of Medicine, 101 Daehak-ro, Jongno-gu, Seoul, 03080 Republic of Korea; 6 Institute of Medical and Biological Engineering, Medical Research Center, Seoul National University College of Medicine, 1 Gwanak-ro, Gwanak-gu, Seoul, 08826 Republic of Korea

**Keywords:** Stroke, Biomedical engineering, Dysphagia

## Abstract

Identification of prognostic factors for swallowing recovery in patients with post-stroke dysphagia is crucial for determining therapeutic strategies. We aimed at exploring hyoid kinematic features of poor swallowing prognosis in patients with post-stroke dysphagia. Of 122 patients who experienced dysphagia following ischemic stroke, 18 with poor prognosis, and 18 age- and sex-matched patients with good prognosis were selected and retrospectively reviewed. Positional data of the hyoid bone during swallowing were obtained from the initial videofluoroscopic swallowing study after stroke onset. Normalized hyoid profiles of displacement/velocity and direction angle were analyzed using functional regression analysis, and maximal or mean values were compared between the good and poor prognosis patient groups. Kinematic analysis showed that maximal horizontal displacement (P = 0.031) and velocity (P = 0.034) in forward hyoid motions were significantly reduced in patients with poor prognosis compared to those with good prognosis. Mean direction angle for the initial swallowing phase was significantly lower in patients with poor prognosis than in those with good prognosis (P = 0.0498). Our study revealed that reduced horizontal forward and altered initial backward motions of the hyoid bone during swallowing can be novel kinematic features indicating poor swallowing prognosis in patients with post-stroke dysphagia.

## Introduction

Stroke is one of leading causes of death and disabilities worldwide^1^. Post-stroke dysphagia is a common complication following stroke^2^. It can induce malnutrition, dehydration, and aspiration pneumonia, which can potentially increase mortality or the duration of hospitalization, and can interfere with the recovery of the neurological and functional status of patients^3–5^. Although most patients with post-stroke dysphagia follow a benign clinical course, 13–18% can show severe dysphagia that can persist for more than 6 months^6,7^. Identification of prognostic factors for poor swallowing recovery in patients with post-stroke dysphagia is crucial for determining therapeutic strategies and counseling patients and their relatives.

Kinematic analysis of videofluoroscopic swallowing study (VFSS) has contributed to the exploration of novel swallowing characteristics by investigating motions of swallowing-related structures. Numerous studies have reported that the pharyngeal swallowing motions were disorganized in dysphagia patients with varying etiologies^3,5,8–11^. In particular, the hyoid bone, which is a ‘U’-shaped structure located in anterior neck^12^, has been utilized as a target structure in previous kinematic studies, since hyoid motions are the main physiological events that occur during pharyngeal swallowing^13–15^, and the hyoid bone can be well-visualized in fluoroscopic images^16^. It moves anteriorly and superiorly in normal swallowing, actively opening the upper esophageal sphincter and leading to an epiglottic tilt; this is the principal protection mechanism of pulmonary aspiration^17,18^. It is known that forward movements of the hyoid bone are substantially decreased in patients with post-stroke dysphagia, which is one of the distinctions compared with healthy individuals^19^. However, there have been rare studies that have investigated hyoid kinematic features associated with swallowing recovery in patients with post-stroke dysphagia.

Functional linear regression analysis (FLR) is a version of linear regression analysis where dependent or independent variables are functional^20^. It is used to analyze the functional data that are converted from discrete observations in the form of a time series. This is done by using basis function expansion and smoothing methods, thus enabling the quantitative analysis of time-series data for group comparison showing regression coefficient functions and differential time interval^21^. Previous swallowing kinematic studies have analyzed swallowing motions by observing discrete variables such as maximum or mean displacement, or velocity rather than the entire profile of movements over time^11^. However, this traditional approach may be limited in exploring novel kinematic features of pathologic swallowing because it does not consider pointwise differences in hyoid trajectories over time. In this regard, FLR allows the collection of time-series data of the hyoid bone during the swallowing process to be represented in new ways than the previously described ones; these data can be beneficial for analyzing group differences in a quantitative manner^22^.

Although the prognostic features of post-stroke dysphagia should receive attention in clinical practice, hyoid kinematic features for swallowing prognosis have not been fully elucidated. FLR can be a beneficial method to compare time-series motion data. In this study, we used FLR and aimed to investigate novel hyoid kinematic features for poor swallowing prognosis in patients with post-stroke dysphagia.

## Methods

### Acquisition of clinical and VFSS data

Clinical and VFSS data of consecutive patients who experienced an acute ischemic stroke and were referred for VFSS due to dysphagia between January 1, 2014, and June 31, 2018 were retrospectively obtained^23^. The exclusion criteria were as follows: concomitant neurologic diseases associated with dysphagia, age less than 19 years, tracheostomy, unconsciousness, history of dysphagia, no swallowing reflex on VFSS, VFSS images of poor quality, and no records of brain magnetic resonance imaging. After exclusion, a total of 122 patients with post-stroke dysphagia were included in the kinematic analysis of VFSS. Among these candidates, 24 (17.5%) patients showed poor swallowing recovery until 6 months after stroke onset. Age- matching and sex-matching were performed between patients with good and poor prognosis for swallowing function because age and sex may affect the kinematic parameters of the hyoid bone during swallowing^8,13,24^. Finally, 18 patients with poor prognosis of swallowing function (no recovery to pre-stroke status at 6 months) and 18 age- and sex-matched patients with good prognosis of swallowing function (recovery to pre-stroke status at 6 months) were selected for this study.

Clinical data regarding age, sex, stroke severity (mild, 0–6; moderate, 7–16; severe, 17–40) in terms of the National Institute of Health Stroke Scale upon admission^25^, stroke location, vascular territory of the brainstem^26,27^ stroke laterality, multifocal lesions, bilateral lesions at the corona radiata, basal ganglia and/or internal capsule, severity of white matter hyperintensity in terms of the Fazekas rating scale (mild, $\ge$ 5; moderate-to-severe, < 5)^28^, clinical dysphagia scale score (mild, < 20; moderate-to-severe, $\ge$ 20)^29^, recommended tube feeding at the initial VFSS, and duration between stroke onset and the initial VFSS were obtained. Good or poor prognosis of swallowing function was defined based on the feeding status, which necessitates tube placement or diet modification at 6 months after the onset of stroke according to follow-up VFSS or clinical judgments in outpatient clinics.

**Figure 1:**
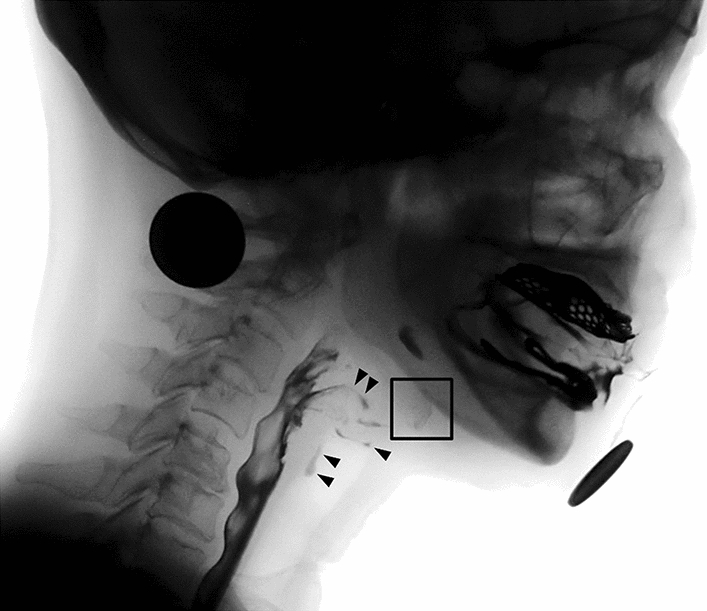
The hyoid bone (black square) observed in the image of videofluoroscopic swallowing study. Liquid bolus was aspirated in the airway (arrow head).

All patients in this study were referred to physiatrists for swallowing assessments and were scheduled to undergo VFSS if post-stroke dysphagia was suspected. VFSS was performed by physiatrists to assess swallowing function and establish feeding strategies. Two-dimensional X-ray images were obtained from patients with post-stroke dysphagia by using VFSS to assess hyoid motions during swallowing in the lateral view (Fig. 1). During VFSS, patients were instructed to swallow various liquid and solid foods mixed with a barium solution, which was prepared by using a standardized method for swallowing evaluation. In this study, only VFSS images obtained after using 2 mL of a 35% w/v diluted barium solution (Solutop Suspension, Tae Joon Pharm Corp., Ltd., Seoul, Korea) were included for kinematic analysis because kinematic parameters of the hyoid bone depend on the volume and viscosity of the liquid. The sampling frequency of VFSS images was 30 images/s.

### Swallowing kinematic analysis

All VFSS images were acquired from the patients included in this study. To extract positional data of the hyoid bone from these images, the swallowing motion analysis software called the spatio-temporal analyzer for motion and physiologic study (STAMPS; https://github.com/cmookj/stamps) was used in the present study^30^. The positional data of the second cervical vertebrae (C2) and the fourth cervical vertebrae (C4), and a coin with a diameter of 23.5 mm as a reference object were additionally obtained to adjust neck movements during swallowing and to normalize the length of displacement. A local coordinate system was applied for each VFSS image in which the origin and the vertical axis were defined as the anteroinferior vertex of the C4 and the line connecting the origin and the anteroinferior vertex of the C2. The starting point of the hyoid bone was set as the origin of the coordinate axes for comparison of trajectories. Previously published researches have provided detailed information about methods for determining the relative position and time lapse in the motion of the hyoid bone during swallowing^11, 30^.

**Figure 2:**
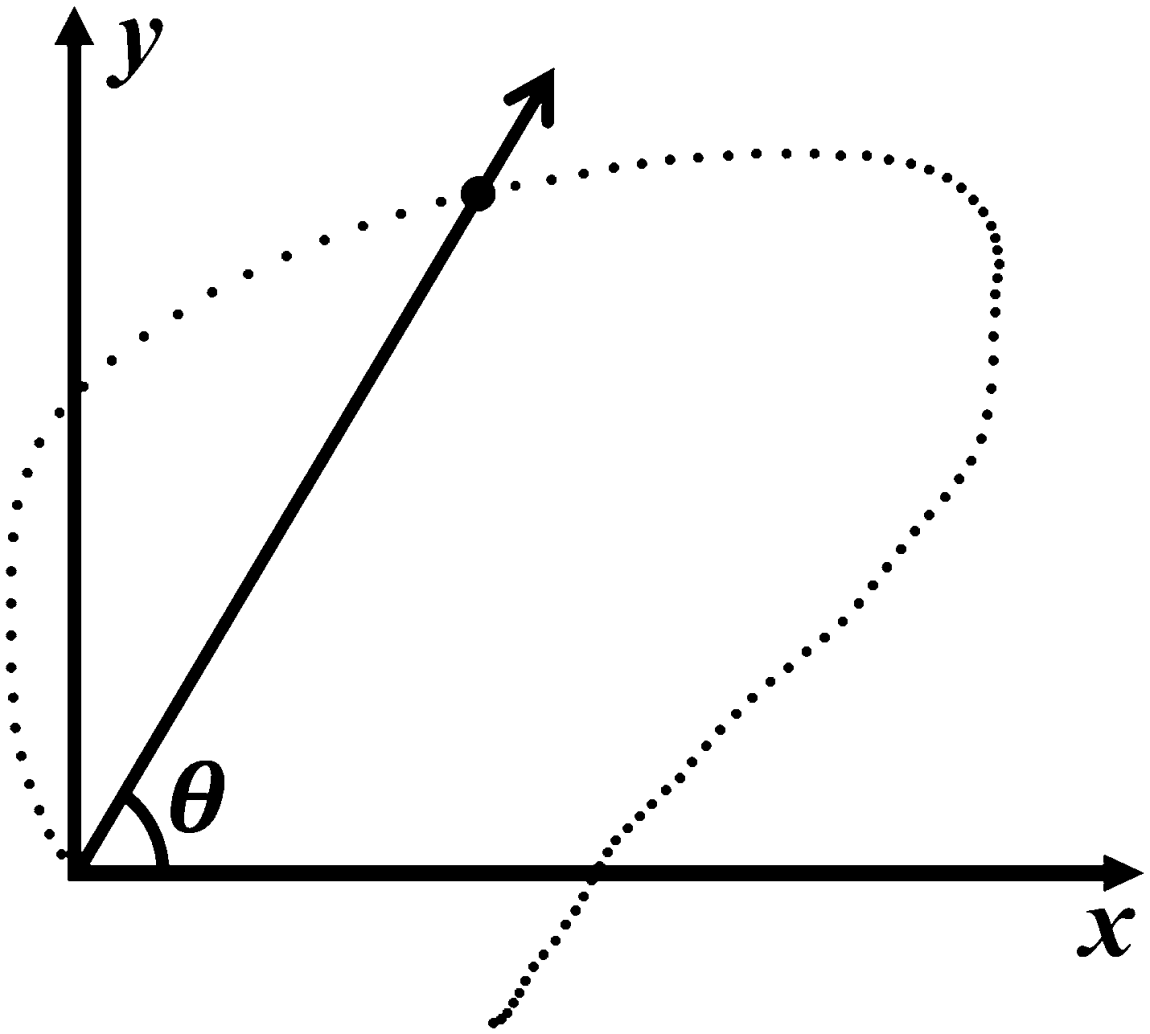
An illustration to show a direction angle of the hyoid bone during swallowing. The direction angle was defined as an angle that characterizes the direction of a straight line for the hyoid bone with respect to a positive horizontal axis.

The time between the start and end points of the swallowing process was interpolated as values from 0 to 100 for temporal normalization^31^. The starting point was defined as the initiation of hyoid motion that resulted in a swallow or when the hyoid bone was located at the lowest position during swallowing^32^. The terminal point was defined as the termination of hyoid bone motion after swallowing. Horizontal and vertical motions of the hyoid bone were defined as the motions along the horizontal and vertical axes. An angle that characterizes the direction of a straight line for the hyoid bone with respect to a positive horizontal axis was defined as the hyoid direction angle (Fig. 2). In this study, the 5th–20th percentile duration was defined as the early phase of swallowing to calculate the hyoid direction angle due to its high variability until the 5th percentile duration.

### Functional regression analysis

Positional data of the hyoid bone were converted to functional data after adjustment for neck movements and normalization. Discrete observations in the *i*th subject, ${W}_{i}$, were transformed to a linear combination of basis spline functions, ${\phi }_{\mathrm{k}}$. The given true functions of ${X}_{i}(t)$ and observation value with noise, ${W}_{i}\left(t\right)$, are represented as ${W}_{i}\left(t\right)={X}_{i}\left(t\right)+{\epsilon }_{i}(t)$ at time point *t*. ${X}_{i}(t)$ is estimated as $\widehat{{X}_{i}}\left(t\right)= \sum_{k=1}^{K}{\widehat{c}}_{ik}{\phi }_{k}(t)$ using a basis expansion, in which the ${\widehat{c}}_{i1}$,…,${\widehat{c}}_{ik}$ are the basis coefficients and determine the relative weights of each basis spline function in constructing the built curve for curve *i*^33^. In this study, the response, ${y}_{i}(t)$, is functional and the predictors ${z}_{ij}$ are bivariate including groups with good and poor swallowing prognosis. The response function $y$ can be represented as ${y}_{i}\left(t\right)= \sum {{\varvec{Z}}}_{{\varvec{i}}{\varvec{j}}}{\beta }_{j(t)}+ {\varepsilon }_{i(t)},$ in which the regression coefficients ${\beta }_{j(t)}$ are a function of time and indicate how the intergroup differences change at each point in time ($t$)^33^. Roughness penalties were utilized for smoothing and regularization to prevent overfitting. Generalized cross-validation was used to determine the lambda value and the number of basis functions to be used for penalization. In the current study, velocity was approximated using the symmetric difference quotient as the sequence of the finite differences of the displacement^11^. For all measures along time *t*, the difference in hyoid motion was considered to be significant when the 95% confidence intervals of the regression coefficients did not present with a value of zero. Statistical significance was set at P < 0.05. The FLR process was conducted using R version 3.5.2 (The R Foundation, Vienna, Austria) with the FDA package. The two-dimensional hyoid trajectories were illustrated using MATLAB R2019a (The Mathworks, Natick, MA, USA).

The clinical information was compared between patients with post-stroke dysphagia who showed good and poor swallowing prognosis using an independent-sample *t*-test for continuous variables, and the chi-square test or Fisher’s exact test for categorical variables. The maximal values of hyoid displacement (HD) and velocity (HV) and mean values of direction angle for the 5th–20th percentile durations were compared between the age- and sex-matched stroke patients, using an independent-sample $t$ test or the Mann–Whitney U test. Statistical analyses were conducted using SPSS software (version 19; SPSS Inc., Chicago, IL, USA), and the significance level was set at P < 0.05. This study was approved by the Institutional Review Board of Seoul National University Hospital (IRB No. 1707-178-875) and the need to obtain informed consent was waived by Institutional Review Board of Seoul National University Hospital due to the retrospective nature of the study. It was performed in accordance with all relevant guidelines and regulations.

## Results

### Clinical characteristics and matching

Table 1 shows the clinical characteristics of age- and sex-matched stroke patients with good and poor prognosis for swallowing function. The mean age of the two groups was not significantly different (good prognosis, 73.9 ± 8.9; poor prognosis, 73.6 ± 8.9; P = 0.912). Among the clinical and radiologic variables, recommended tube feeding at initial VFSS was the only factor that differed significantly between the two groups (good prognosis, 1 (5.6%); poor prognosis, 14 (77.8%); P < 0.001).

**Table 1:** Clinical characteristics of age- and sex-matched stroke patients with good and poor prognosis for swallowing function (n = 36).

	Good prognosis (n = 18)	Poor prognosis (n = 18)	*P*
Age	73.9 ± 8.9	73.6 ± 8.9	0.912
**Sex**			1.000
Male	12 (66.7)	12 (66.7)	
Female	6 (33.3)	6 (33.3)	
**NIHSS at admission**			0.274
Mild (0–6)	6 (35.3)	11 (61.1)	
Moderate (7–16)	9 (52.9)	5 (27.8)	
Severe (17–40)	2 (11.8)	2 (11.1)	
**Vascular territory of brainstem**			
Anteromedial territory	0 (0.0)	3 (16.7)	0.229
Anterolateral territory	1 (5.6)	5 (27.8)	0.177
Lateral territory	1 (5.6)	3 (16.7)	0.603
Posterior territory	0 (0.0)	0 (0.0)	–
**Lesion laterality**			0.131
Right	7 (38.9)	3 (16.7)	
Left	7 (38.9)	13 (72.2)	
Bilateral	4 (22.2)	2 (11.1)	
**Lesion location**			
Frontal lobe	8 (44.4)	3 (16.7)	0.146
Parietal lobe	6 (33.3)	3 (16.7)	0.443
Temporal lobe	4 (22.2)	4 (22.2)	1.000
Occipital lobe	4 (22.2)	1 (5.6)	0.338
CR	7 (38.9)	9 (50.0)	0.502
BG/IC	6 (33.3)	9 (50.0)	0.310
Insula	2 (11.1)	5 (27.8)	0.402
Thalamus	2 (11.1)	2 (11.1)	1.000
Midbrain	0 (0.0)	0 (0.0)	–
Pons	1 (5.6)	3 (16.7)	0.603
Medulla oblongata	1 (5.6)	4 (22.2)	0.338
Cerebellum	4 (22.2)	1 (5.6)	0.338
Multifocal lesions	3 (16.7)	0 (0.0)	0.229
Bilateral lesions at CR/BG/IC	4 (22.2)	9 (50.0)	0.164
Severe white matter hyperintensities	1 (5.6)	6 (33.3)	0.088
Clinical dysphagia scale $\ge$ 20	7 (38.9)	11 (61.1)	0.182
Recommended tube feeding at initial VFSS	1 (5.6)	14 (77.8)	< 0.001*
Duration between stroke onset and the initial VFSS	12.9 ± 6.1	16.2 ± 7.1	0.126

BG, basal ganglia; CR, corona radiata; IC, internal capsule; NIHSS, National Institutes of Health Stroke Scale; VFSS, videofluoroscopic swallowing study.

Values are presented as mean ± standard deviation or number (percent).

*P-value < 0.05.

### Functional regression analysis for displacement, velocity, and direction angle of the hyoid bone

**Figure 3:**
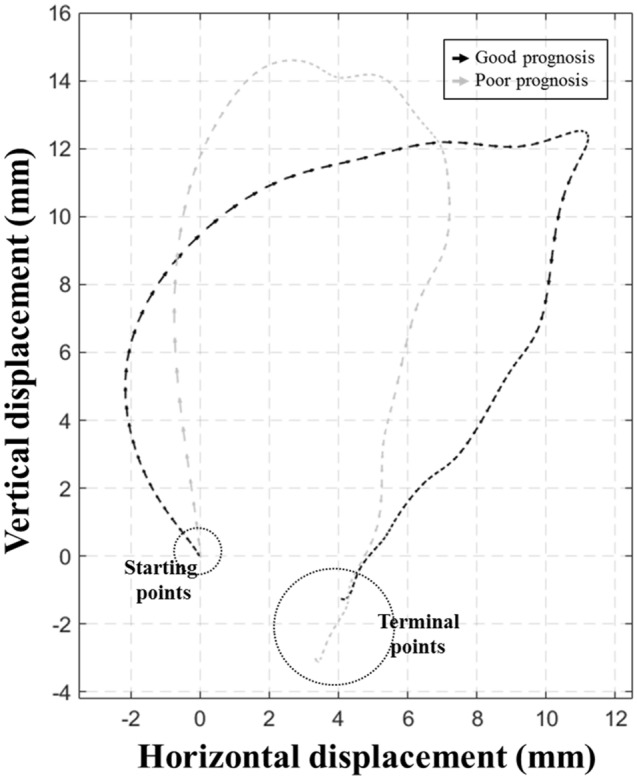
The mean two-dimensional trajectories of the hyoid bone during swallowing in age- and sex-matched stroke patients with good and poor prognosis for swallowing function.

**Figure 4:**
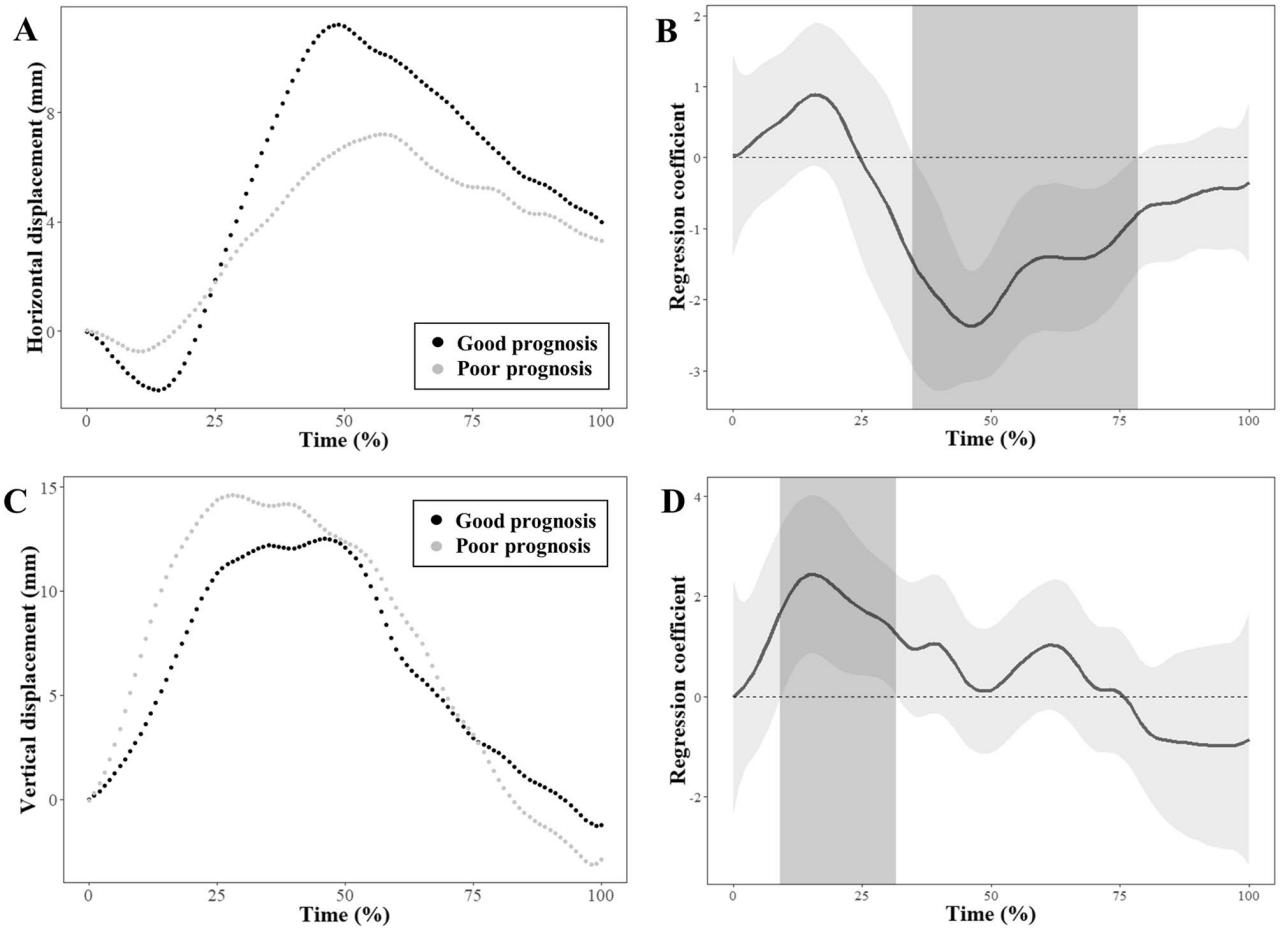
Results of functional regression analysis for horizontal and vertical hyoid displacements. Mean trajectories are illustrated for the horizontal (**A**) and vertical (**C**) displacements in age- and sex-matched stroke patients with good and poor prognosis. Estimated regression coefficient functions with 95% confidence intervals are shown for the horizontal and vertical displacements between stroke patients with good and poor prognosis (**B**,**D**). The light gray zones denote the time interval where the mean difference is significant between the groups.

Figure 3 shows the mean two-dimensional trajectories of the hyoid bone during swallowing in the age- and sex-matched stroke patients with good and poor prognosis for swallowing function. The mean HDs in the horizontal and vertical planes in the two groups are shown in Fig. 4A,C. The regression coefficient functions representing intergroup differences for the horizontal and vertical HDs over time between the two groups are shown in Fig. 4B,D. Horizontal HD differed significantly between the patients with good and poor prognosis over the forward motions (35th–78th percentiles). Vertical HD showed a significant difference over time between the two groups over the upward motions (9th–32nd percentiles).

**Figure 5:**
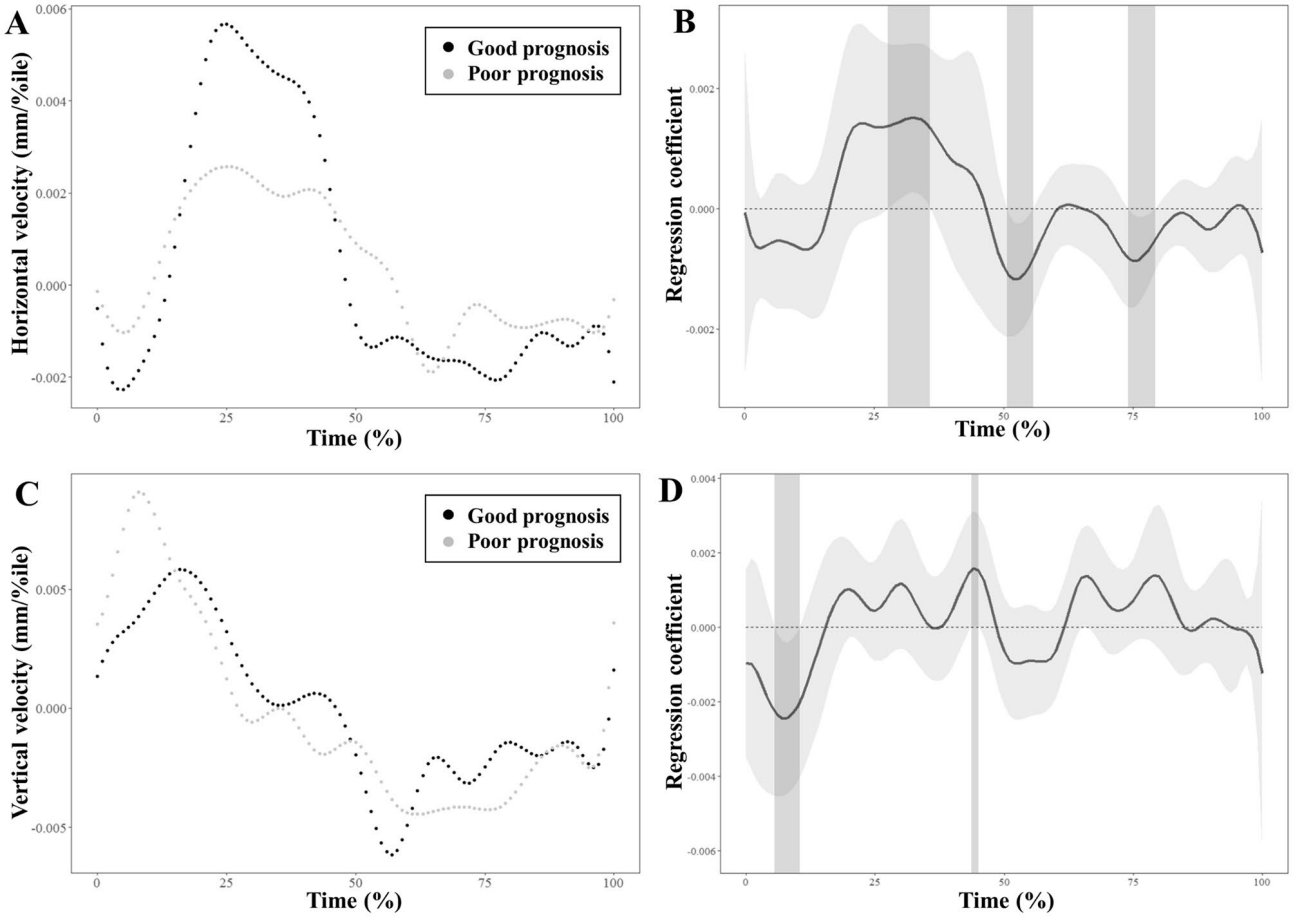
Results of functional regression analysis for horizontal and vertical hyoid velocities. Mean trajectories are illustrated for the horizontal (**A**) and vertical (**C**) hyoid velocities in age- and sex-matched stroke patients with good and poor prognosis. Estimated regression coefficient functions with 95% confidence intervals are shown for the horizontal (**B**) and vertical (**D**) velocities between stroke patients with good and poor prognosis. The light gray zones denote the time interval where the mean difference is significant between the groups.

The mean HVs in the horizontal and vertical planes for the age- and sex-matched stroke patients with good and poor prognosis for swallowing function are shown in Fig. 5A,C. The regression coefficient functions representing intergroup differences for the horizontal and vertical HVs over time between the two groups are shown in Fig. 5B,D. The HV showed significant differences between the patients with good and poor prognosis at the 28th–36th, 51st–56th, and 74th–79th percentiles in the horizontal plane, and the 5th–11th and 44th–45th percentiles in the vertical plane.

**Figure 6:**
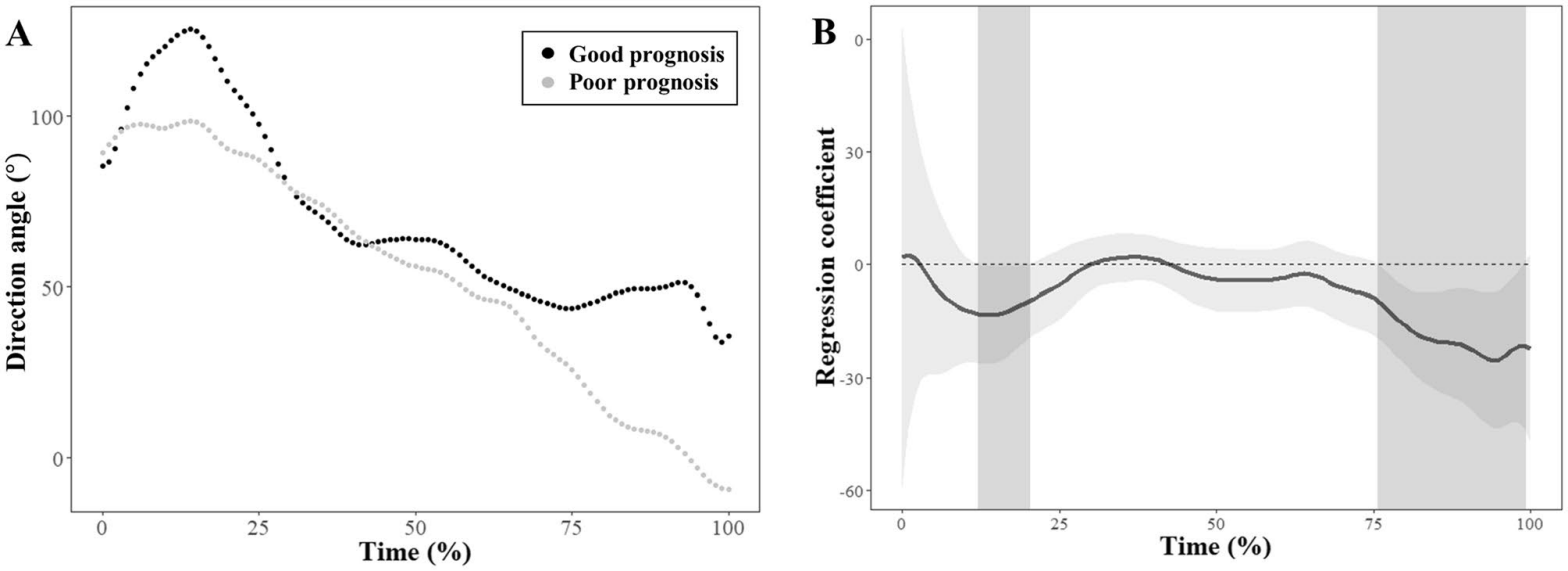
Results of functional regression analysis for hyoid direction angles. Mean trajectories are illustrated for the direction angles in age- and sex-matched stroke patients with good and poor prognosis (**A**). Estimated regression coefficient functions with 95% confidence intervals are shown for the direction angles between stroke patients with good and poor prognosis (**B**). The light gray zones denote the time interval where the mean difference is significant between the groups.

The mean trajectories for the hyoid direction angles in the age- and sex-matched stroke patients with good and poor prognosis for swallowing function are illustrated in Fig. 6A. The regression coefficient function representing intergroup differences over time between the two groups is shown in Fig. 6B. The hyoid direction angle showed significant differences between the patients with good and poor prognosis at the 12th–19th and 76th–99th percentiles.

### Analysis for maximal values of hyoid kinematic parameters

Table 2 shows the results of analysis for the maximal values of HD and HV, and the mean direction angle for the 5th–20th percentile duration. Both maximal horizontal HD (P = 0.031) and HV (P = 0.034) in the forward motions differed significantly between the two groups. The mean direction angle for the 5th–20th percentile duration was also significantly different between the two groups (P = 0.0498).

**Table 2:** Results of analysis for the maximal and mean kinematic parameters of hyoid motion in age- and sex-matched stroke patients with good and poor prognosis (n = 36).

	Good prognosis (n = 18)	Poor prognosis (n = 18)	*P*
Total swallowing duration (s)	1.76 ± 0.45	1.89 ± 0.47	0.403
**Maximal horizontal displacement (mm)**			
Backward	− 3.49 ± 2.59	− 3.01 ± 3.36	0.282
Forward	12.43 ± 4.81	9.22 ± 3.95	0.031*
**Maximal vertical displacement (mm)**			
Upward	15.37 ± 7.14	16.81 ± 7.10	0.548
**Time to maximal displacement (percentile)**			
Horizontal, backward	17.38 ± 7.45	17.14 ± 14.53	0.950
Horizontal, forward	48.96 ± 8.69	47.98 ± 13.76	0.799
Vertical, upward	36.73 ± 13.26	31.87 ± 10.44	0.230
**Maximal horizontal velocity (mm/percentile)**			
Backward	− 0.63 ± 0.41	− 0.56 ± 0.38	0.393
Forward	1.28 ± 0.50	0.96 ± 0.43	0.034*
**Maximal vertical velocity (mm/percentile)**			
Upward	1.36 ± 0.52	1.76 ± 0.98	0.114
**Time to maximal velocity (percentile)**			
Horizontal, backward	25.36 ± 22.24	16.02 ± 17.60	0.173
Horizontal, forward	29.21 ± 8.25	25.64 ± 14.53	0.372
Vertical, upward	19.20 ± 10.97	13.81 ± 10.83	0.071
Mean direction angle for 5th–20th percentile duration	119.18 ± 38.19	96.45 ± 33.91	0.0498*

Values are presented as mean ± standard deviation.

*P-value < 0.05.

## Discussion

The current study aimed to investigate the characteristic kinematic parameters of the hyoid bone, which are associated with poor swallowing prognosis in patients with post-stroke dysphagia. Pointwise differences in swallowing motion over time were analyzed between patients with good and poor swallowing prognosis by using FLR and analyzing maximal or mean values of hyoid kinematic parameters. The results showed that HD and HV of the horizontal plane during the forward motions were significantly reduced in patients with poor swallowing prognosis compared with those with good swallowing prognosis, though the values of the vertical plane were not significantly different. Decreased direction angle of the hyoid bone during the early phase of swallowing was also evident in patients with poor swallowing prognosis.

This study suggests that a decrease in horizontal HD and HV indicates poor swallowing prognosis in post-stroke dysphagia. HD and HV, which were discrepant in comparison between stroke patients and healthy controls in a previous study^19^, were consistently decreased in stroke patients with poor swallowing prognosis compared to those with good swallowing prognosis in the current study. Horizontal HV increased and then decreased rapidly in stroke patients with good prognosis compared to those with poor prognosis according to the results of FLR analyses. A substantial decrease in horizontal HD and HV at the early phase of stroke may reflect initial severity of dysphagia which has been previously reported to be associated with the hindrance of swallowing recovery^23,34–36^. Since the contraction sequence of the hyoid-adjacent muscles is constant during the pharyngeal phase of swallowing^37^, damage to the swallowing-related neural systems that control the hyoid-adjacent muscles can cause the deterioration of this sequence^38,39^. Decreased forward motion of the hyoid bone caused by impairment of the swallowing-related neural systems can result in poor relaxation of the upper esophageal sphincter, accumulation of the residue in the pyriform sinus, and an increase in aspiration risk^18^. In addition, hyoid motions during the late phase of swallowing are involved in returning to the initial position before swallowing and can be essentially affected by the hyoid trajectories in the early phase of swallowing. Thus, hyoid trajectories during the late phase of swallowing can be an indirect result of weakness of the hyoid-adjacent muscles after stroke and clinical emphasis should be placed on the early phase of swallowing rather than on the late phase.

The direction angle, which is the angle of the vectors, was adopted in this study as a kinematic parameter to reflect the dominant direction of the hyoid motions in the early phase of swallowing. Figure 3 shows that the vertical direction during backward hyoid motion is prominent in stroke patients with poor swallowing prognosis compared with those with good swallowing prognosis. The results of the statistical analyses showed that vertical HD and HV did not differ significantly between the two groups. Rather, the vertical HD and HV were greater in the FLR analysis and the direction angle in the early phase of swallowing was less in patients with poor prognosis than those with good prognosis. These findings suggest that the vector of the hyoid motion in the early phase of swallowing is more vertical than horizontal in patients with poor swallowing prognosis. The disjunction between vertical and horizontal hyoid motions is grossly consistent with the findings of previous studies assessing dysphagia patients^8,11,40^. The decreased direction angle can be explained by weakness of the hyoid-adjacent muscles to pull the hyoid bone posteriorly, including the stylohyoid muscle and posterior digastric muscle^37^. The capacity to generate strong forces in the forward direction by lengthening the suprahyoid muscles can be deteriorated in the vertical direction of backward force vectors during the early phase of swallowing in patients with poor prognosis of swallowing function^11^. Additionally, the observation of preserved vertical motions during swallowing was in concordance with the findings of a previous research which showed that maximal HV was preserved in the vertical plane, but decreased significantly in the horizontal plane in stroke patients with aspiration, compared with healthy participants^19^. In this previous study, maximal HD was preserved in the vertical plane unlike that in the horizontal plane, even though the difference between the two groups was not significant. Another study comparing healthy younger and older participants also indicated that vertical HD was greater in older participants than in younger participants^8^. Effortful swallowing is a plausible compensatory mechanism to explain the following disjunction: HD and HV in the horizontal plane are less affected by effortful swallowing than the HD and HV in the vertical plane^41^. To offset the weakness of the pharyngeal musculature for swallowing difficulty, patients with more severe dysphagia may show forceful swallowing as a natural compensation^42^.

In this study, FLR was used as the main statistical method to identify pointwise differences in swallowing trajectories over time between stroke patients with good and poor prognosis for swallowing function. This method was previously used to explore novel kinematic features related to swallowing by quantitatively analyzing hyoid trajectories in patients with Parkinson’s disease^11^. The present study showed a reduced direction angle in the early phase of swallowing, which was identified using FLR by analyzing time-series data for the hyoid bone during swallowing. This finding supports the relative preservation of hyoid motions in the vertical plane, contrary to hyoid motions in the horizontal plane. In fact, previous studies investigating the effects of therapeutic interventions on swallowing outcomes in patients with post-stroke dysphagia usually did not adopt kinematic parameters to determine patient inclusion^43^. This is possibly because of the technical difficulty in conducting image processing of VFSS data and analyzing kinematic parameters related to swallowing in clinical studies. The methods used to quantitatively analyze swallowing kinematics in this study can be potentially useful for reducing or correcting a potential selection bias in further comparative studies (e.g., randomized controlled trials) on post-stroke dysphagia.

This study has several limitations. First, the sample size of the current study was small. To analyze the differences between good and poor swallowing prognosis groups, age- and sex-matching were used; many patients with good swallowing prognosis were not included in the analysis. It was difficult to conduct subgroup analyses to identify subtle between-group differences in clinical and radiologic variables. This is the first study to explore swallowing kinematic features of stroke patients with poor prognosis and future studies involving a larger sample size may be necessary to generalize the results of this study. Second, hyoid motions for only the specific liquid volume were analyzed to ensure comparability in this study. Previous studies have indicated that viscosity and volume of the ingested liquid can affect the hyoid kinematic features considerably. Hyoid kinematic analyses for diverse viscosity or volume of liquid can be helpful to explore novel kinematic features indicating poor swallowing prognosis of post-stroke dysphagia in further studies. Third, potential confounding factors such as sarcopenia and nutritional status, which can affect swallowing function, were not measured in this study and should be considered in prospective comparative studies for swallowing interventions.

In conclusion, the swallowing kinematic analysis based on FLR indicated reduced horizontal HD and HV and decreased direction angle for the early phase of swallowing in patients with post-stroke dysphagia showing poor swallowing prognosis. Altered initial backward motions and reduced horizontal forward motions of the hyoid bone during swallowing may be novel kinematic features for poor prognosis of post-stroke dysphagia. FLR can potentially provide new insights in understanding swallowing kinematics and physiology in pathologic swallowing in further studies.

## Data Availability

The datasets for hyoid motions generated during and/or analyzed during this study are included in this published article.
